# A novel technique for prevention of anterior fistula and facilitation of alveolar cleft repair: Gingivoperiosteoplasty with palatoplasty

**DOI:** 10.12669/pjms.38.7.5422

**Published:** 2022

**Authors:** Owais Ahmed, Sobia Yasmeen, Muhammad Imran Khan, Mirza Shehab Afzal Beg

**Affiliations:** 1 Dr. Owais Ahmed, MBBS. Department of Plastic and Reconstructive Surgery, Liaquat National Hospital and Medical College, Karachi, Pakistan; 2 Dr. Sobia Yasmeen, FCPS. Department of Plastic and Reconstructive Surgery, Liaquat National Hospital and Medical College, Karachi, Pakistan; 3 Dr. Mohammad Imran Khan, MBBS. Department of Plastic and Reconstructive Surgery, Liaquat National Hospital and Medical College, Karachi, Pakistan; 4 Prof. Mirza Shehab Afzal Beg, FRCS (PLAST). Department of Plastic and Reconstructive Surgery, Liaquat National Hospital and Medical College, Karachi, Pakistan

**Keywords:** Gingivoperiosteoplasty, Palatoplasty, Anterior fistula, Bone growth, Secondary bone grafting

## Abstract

**Background & Objectives:**

The Cleft palate is one of the most commonly encountered congenital deformity in plastic surgery clinics and can be associated with cleft lip and alveolus. Though palate repair can be associated with several complications, the most frequent and troublesome is anterior fistula formation. Various technical modifications are in practice to avoid this dreaded complication. We have started combining gingivoperiosteoplasty with palate repair to avoid postoperative anterior fistula formation and to close alveolar cleft at the same time.

**Methods:**

A prospective study was performed at the department of plastic and reconstructive surgery, Liaquat National Hospital, Karachi and selected patients were enrolled in the study after informed consent. A total of 15 patients were operated on from January 2017 to December 2020. All patients had cleft palate repair along with primary gingivoperiosteoplasty (GPP) at the age of standard palatal repair. Buccal/oral and nasal layers of the alveolus were dissected as per standard gingivoperiosteoplasty and repaired in continuation with nasal and oral layers of the palate. Postoperatively, the standard cleft palate repair protocol was followed. Follow-up was done at four weeks, 12 weeks, and six months and repair integrity was checked. Future follow-up at 4-5 years of age is planned to see the effect on alveolar collapse, bone growth, and the need for secondary bone grafting.

**Results:**

All patients were followed up regularly. None had a complication of fistula. The repairs of both palate and alveolus remained intact. Patients were kept on the follow-up to assess the need for alveolar bone grafting in the future.

**Conclusion:**

Gingivoperiosteoplasty combined with the palatal repair is a novel technique for the prevention of anterior palatal fistula.

## INTRODUCTION

The Cleft palate is one of the most commonly encountered congenital deformity in plastic surgery clinics with an incidence of 1.91 per 1000 births in Pakistan.^1^ In the cleft palate, surgery, the primary objective is to regain normal function of speech and separation of oral-nasal cavities. Several techniques have been proposed and implied over time, but only a few have stood the test of time.^2^,^3^ The palate is repaired traditionally with interrupted sutures between the ages of 6 and 18 months.^4^

Though palate repair can be associated with many complications, including respiratory distress, infections, dehiscence, and the most frequent and troublesome, anterior fistula formation with a recently reported incidence of 8.6%.^5^ In past literature, the reported incidence of fistula formation after cleft palate repair varies between 5% and 34%.^6^ For the patients having cleft alveolus with cleft lip and palate, many options are available including primary bone grafting, secondary bone grafting, and gingivoperiosteoplasty.^7^ Gingivoperiosteoplasty (GPP) is usually done at the time of lip repair (3- 6 months of age) while the cleft alveolus is very small.^8^ Power and Matic reported that when the palatal repair was done after primary GPP at the time of traditional lip repair, approximation of the anterior edge of the palate with the posterior edge of the repaired alveolus becomes technically difficult due to poor visualization that can lead to fistula formation.^9^ Various technical modifications are in practice to avoid this dreaded complication.^10^ In a Study, Losquardo et al.^7^ described a regimen of performing gingivoperiosteoplasty at 12 months with palatal repair and reported good outcomes in terms of reduction or elimination for secondary bone graft and decreases the incidence of anterior fistula following palatal repair.

In our center, we have started the technique of combining gingivoperiosteoplasty with palate repair rather than lip repair because that at time of lip repair we give back cuts in gingiva-buccal sulcus for tension free lip repair therefore, we cannot harvest buccal lining of upper alveolus adjacent to cleft for GPP. We are sharing our experience of doing GPP at the time of palate repair to avoid alveolar collapse with growing age.

## METHODS

A prospective descriptive study was performed at the department of plastic and reconstructive surgery, Liaquat National Hospital, Karachi from January 2017 to December 2020. Ethical approval was taken from the institutional review board with reference number 0606-2020-LNH-ERC and the informed consent was obtained from the patient’s parents and they were briefed about the nature and purpose of the study and possible outcomes. Patients with unilateral complete cleft lip and palate with cleft alveolus, from 8 to 18 months of age, male/female were included in the study. Patients with age more than 18 months, history of previous palatal surgery, and primary GPP, with associated congenital disorders and syndromes were excluded from the study.

A total of 15 patients were included in the study and were operated on for palatoplasty with gingivoperiosteoplasty. The cleft lip was operated on at the age of 3-5 months by Modified Millard’s technique for all 15 patients in the same center. Information including basic demographics, procedure duration, and complications was documented.

**Fig.1:**
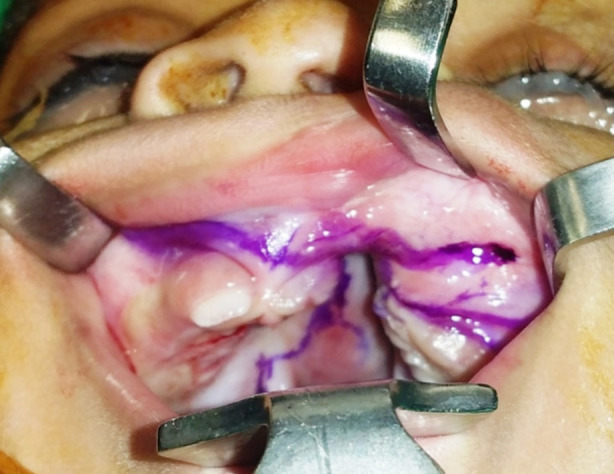
Markings for GPP and palatal repair. Periosteal flaps are marked over the alveolar ridge on the nasal, labial and palatal side.

**Fig.2:**
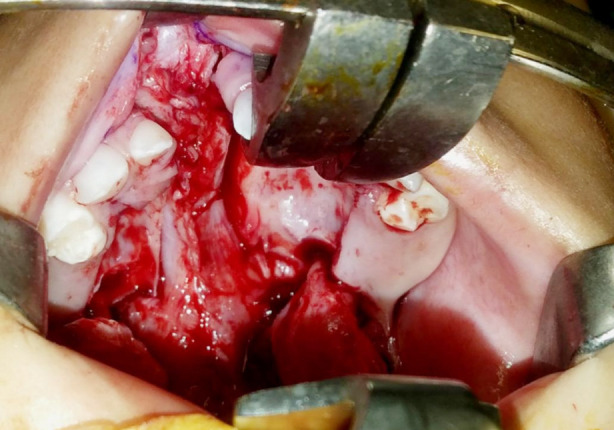
Periosteal and mucosal flaps elevation.

All the procedures were done by a single consultant plastic surgeon with more than 20 years of experience. Under general anesthesia, the patient was kept in a supine position with an extended neck. Marking was done along the incision lines for gingivoperiosteoplasty and palatoplasty (Fig.1). Lidocaine 0.5% with epinephrine was injected along with the markings of the cleft alveolus and cleft palate. The standard palate repair was planned and performed using the Bardach technique^11^ and was combined with gingivoperiosteoplasty i.e., incisions are made along the bilateral margins of the cleft alveolus and pointed toward the nasal spine on the non-cleft side and piriform aperture on the cleft side and connected posteriorly to the nasal and oral layers of the cleft palate. These incisions were joined anteriorly at the apex of the cleft alveolus. Periosteal flaps are raised over the alveolar ridge on the nasal, labial, and palatal sides. The mucosa of the labial vestibule was dissected and released subperiosteally from both sides of the cleft alveolus so that both flaps can be advanced medially (Fig.2).

**Fig.3:**
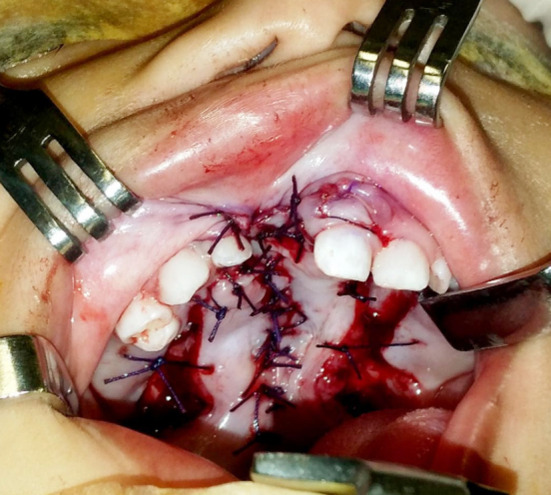
Closure of nasal layer of the alveolus in continuation with the nasal layer of the palate.

**Fig.4:**
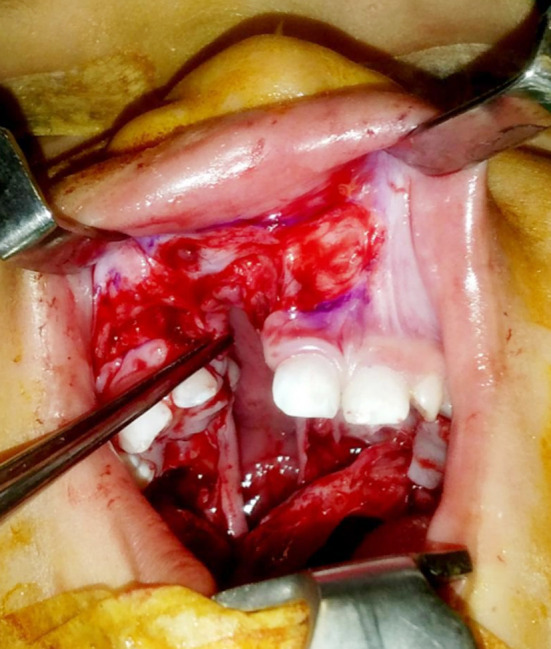
Closure of oral layer of the alveolus in continuation with the oral layer of palate.

The nasal sill of the cleft alveolus was closed in continuation with the nasal layer of the cleft palate (Fig.3) and the oral layer of the palate was then closed and joined with the oral layer of the cleft alveolus by advancing the mucosal flaps of the labial vestibule medially (Fig.4).

Protocols for the standard postoperative care of the cleft palate repair were followed for all patients. Post-operative follow-up was done 1 week, 3 weeks, 3 months, and 6 months and repair integrity was checked. The repair integrity was documented at the 4^th^ and 12^th^ week, and six months At six months, the presence of the anterior fistula was assessed by passing a fine probe either through the nasal cavity or the superior end of the buccal sulcus.^12^ Future follow-up at 4-5 years of age is planned to see the effect on alveolar collapse, bone growth, and the need for additional bone grafting. Data were compiled and analyzed using the Statistical Package for Social Sciences (SPSS) version 25.

## RESULTS

A total of 15 patients were included in the study. There were 10 (66.7%) males and 5 (33.3%) females. The mean age was 10.13 months (range 9-15 months). All the patients had a unilateral cleft of the primary and secondary palate and alveolus. No patient had immediate or early postoperative complications like infection or wound dehiscence. Both palate and alveolus repairs remain intact. All the patients were assessed at six weeks, 12 weeks, and six months postoperatively and none developed postoperative anterior palatal fistula.

## DISCUSSION

The occurrence of cleft lip, palate, and alveolus shows racial predilection, especially in Asians.^6^ The management in terms of early diagnosis and appropriate surgical intervention adds substantially to the health care burden in Pakistan.^13^ A multidisciplinary approach is required for caring for the child with cleft lip, palate, and alveolus that starts with prenatal diagnosis (where available), evaluation for other possible congenital anomalies, decisions about the timing of repair, choice of techniques, and continuing care into adulthood for the secondary procedure when needed.^13^,^14^ Pre and post-operative management of these cleft patients must also include a multidisciplinary team consisting of an otolaryngologist, an orthodontist, and a speech therapist.^10^ Although the prime answer to the care of the cleft patient is surgery some techniques that may improve the surgical outcomes are done in infancy like presurgical nasal-alveolar molding (NAM).^15^

Despite effective and meticulous cleft palate repair the rates of development of palatal fistula are high and affects negatively on the patients’ general health and quality of life due to the presence of symptoms like nasal regurgitation, poor oral hygiene, and nasal emission during speech.^16^ Among all types of postoperative palatal fistulas, anterior fistula is a common complication and occur mostly in individuals who have cleft alveolus along with cleft palate and is a challenging clinical dilemma as the presence of scarring and depth of the palatal arch increased with age and is difficult to approach.^17^ To prevent this cumbersome complication, we started doing palatoplasty with gingivoperiosteoplasty as a single-stage procedure and has excellent results i.e., none of all 15 patients developed postoperative anterior fistula.

Currently, there are three modalities present in the literature for the repair of a cleft alveolus in patients with cleft lip and palate: gingivoperiosteoplasty, primary bone grafting, and Secondary bone grafting.^7^ Gingivoperiosteoplasty initially described by Skoog involves wide dissection on the cleft side of maxillary periosteum to elevate a medially based flap that was rotated subsequently to close the oral and palatal side of the cleft alveolus. He also used surgical (oxidized cellulose) to fill the subperiosteal pocket between the cleft alveolus margins and reported excellent bone growth and maxillary arch stabilization in 52 patients with unilateral or bilateral clefts.^18^

Presently, the most common method for establishing the alveolar continuity is the use of gingivoperiosteoplasty (GPP) during lip repair but the timing of gingivoperiosteoplasty is not much discussed in the literature and very limited data is available.^7^ This procedure is mostly performed at the time of lip repair in most centers that can affect the vertical maxillary growth.^7^ Direct gingivoperiosteoplasty combined with hard palate repair was reported in the literature after cleft lip repair with good outcomes in terms of normal maxillary development and restoration of anatomy.^19^,^20^ William D. Losquadro et al.^7^ performed a single-stage cleft palate repair with direct gingivoperiosteoplasty at one year of age with prior two stage-lip repairs and reported reliable bone growth at the cleft alveolus with the normal eruption of lateral incisor in the majority of the patients. We performed direct gingivoperiosteoplasty combined with cleft palate repair at the age of standardized cleft palate repair with satisfactory preliminary results that are, none of the patients develop anterior fistula or other postoperative complications. The cleft lip repair was performed in a single stage at 3-5 months in our patients.

The cleft lip repair combined with direct gingivoperiosteoplasty has been performed to avoid the need for secondary bone grafting, but also affect the vertical growth of the maxilla.^7^ Direct gingivoperiosteoplasty after presurgical orthopedic i.e., nasal-alveolar molding can produce sufficient bone to avoid the need for secondary bone grafting. In our study, we presented here a novel technique of performing palatoplasty with gingivoperiosteoplasty as a single-stage procedure to prevent the anterior fistula formation, it will induce significant bone growth that can avoid the need for secondary bone grafting. A further follow-up at the age of 4-5 years of age has been planned and the patients will be assessed for bone growth and alveolar collapse by OPG.

### Limitations

It includes small sample size. The role of preoperative nasal-alveolar molding was not assessed in this study along with the size of alveolar cleft is not measured before undergoing GPP and long term follow up is not available at the moment but it is planned to see maxillary growth, teeth eruption and need of secondary alveolar bone grafting.

## CONCLUSION

Gingivoperiosteoplasty combined with palate repair is a simplified technique with multiple benefits including the prevention of anterior palatal fistula as none of our patient in which this combined approach is used developed fistula, while the standard rate of palatal fistula formation is 8.6%.^5^ Further follow-up at the age of 4-5 years is planned to see long term effects of this technique on alveolar collapse, bone growth, and the need for secondary bone grafting.

### Authors’ Contribution:

**SY, OA & MSAB:** Conceived, designed, did statistical analysis & editing of manuscript.

**OA, SY & MIK:** Did data collection, manuscript writing & statistical analysis.

**MSAB & SY:** Did review and final approval of manuscripts. Responsible and accountable for accuracy and integrity of work.
